# Slovenian experience from diagnostic angiography to interventional radiology

**DOI:** 10.2478/raon-2014-0023

**Published:** 2014-11-05

**Authors:** Dusan Pavcnik

**Affiliations:** Dotter Interventional Institute, Oregon Health Sciences University, Portland, U.S.A

**Keywords:** diagnostic angiography, interventional radiology, history

## Abstract

**Background:**

The purpose of writing this article is to document the important events and people in the first 50 years of diagnostic angiography and interventional radiology in Slovenia. During this period not only did the name of the institutions and departments change, but also its governance.

**Conclusions:**

This depicted the important roles different people played at various times in the cardiovascular divisions inside and outside of the diagnostic and interventional radiology. Historical data show that Slovenian radiology has relatively immediately introduced the new methods of interventional radiology in clinical practice.

## Introduction

Diagnostic angiography is the study of blood vessels in humans and animals by x-ray contrast method. To describe its early development we have to go back to the discovery of x-rays.

In 1896, one year after Roentgen’s discovery of x-rays, Haschek and Lindenthal published the first angiogram of an amputated hand using bismuth, lead and barium salts.^1^ The injected contrast mixture demonstrated good absorption of the x-rays and demonstrated the huge potential of this new technique. Of course, a suitable safe contrast material had not been invented yet.

### Egas Moniz, Reynaldo dos Santos, Moses Swick, Werner Forssmann

The Portuguese physician and neurologist Egas Moniz, a Nobel Prize winner in 1949, in 1927 developed carotid angiography by using needle puncture and injection of 22% sodium iodide.^2^ Various forms of carotid angiography remain a fundamental tool, both in diagnosis and interventional procedures on the brain.

Reynaldo dos Santos in 1929, showed that satisfactory opacification of the abdominal aorta and its branches could be obtained by use of translumbar needle and injection of contrast material.^3^ In 1929 Werner Forssmann, Nobel Prize winner in 1956, was the first person to introduce a ureteric catheter into his own heart via antecubital vein (self experiment).^4^ The same year, Moses Swick reported discovery of an opaque organic iodine contrast media that was significantly less toxic than sodium iodide.^5^ It is a remarkable coincidence that these two important radiological developments were reported for the first time in 1929 in the same issue, in the journal Klinische Wochenschrifft: Swick’s paper on the first iodinated water soluble contrast medium, Uroselectan^4^, and Forssmann’s paper on the catheterization of the heart.^5^

The works of Moniz, dos Santos, Swick and Forssmann had great impact on further development of diagnostic angiography.

### Seldinger technique, radioopaque catheters and suitable x ray contrast media

With the introduction of the Seldinger technique in 1953, the procedure became safer and user friendly as no sharp or rigid needles needed to remain inside the vascular lumen. “Catheter replacement of the needle in percutaneous arteriography” was the title of his unique technique published in Acta Radiologica.^6^ With the development of radioopaque thermoplastic catheters (KIFA) in Sweden^7^ and with the availability of suitable intra-arterial contrast media^8^, the groundwork of the modern methods of angiography became well establish. Percutaneous transfemoral transcatheter arteriography was just beginning to emerge in 1950s and in 1960s became the widely applied diagnostic method throughout the world, including Slovenia. Miro Košak introduced the concept of angiography in the Slovenian Journal Zdravstveni Vestnik under the name clinical importance of angiography as diagnostic method in therapy.^9^

### Ivo Obrez and Jože Stropnik

Ivo Obrez, a native of Novo mesto, Slovenia, earned his medical degree from Ljubljana University School of Medicine in 1955. After finishing a residency in radiology in 1961 in Ljubljana, he led the Department of Roentgenology at the General Hospital Novo mesto until 1965. In the early 1960s he spent part of the year at the Universities of Lund and Stockholm, learning angiographic techniques. There he met Herbert Abrams from Stanford University. By the time he moved to Ljubljana on invitation by Prof Stanko Hernja, he had written 3 scientific articles on angiography^10^–^12^, and co-founded this journal, Radiology and Oncology, at the time the journal of *Radiologia Iugoslavica*.^13^ When Ivo Obrez arrived to Ljubljana, he began performing arteriograms, succeeding the surgeon Miro Košak who has done them previously at dislocated fluoroscopic Siemens x ray unit at the Department of Surgery.^9^,^14^ As Ivo Obrez dug into the new tasks, Prof. Hernja and Prof. Košak found resources to support him. The Siemens x-ray apparatus was updated with an image intensifier. With electronic image intensification, the angio procedure could be done in normal light. This led to better visualization of the vascular system and the heart.^12^ Pressure injectors and rapid-change film holders allowed multiple images essential for new studies of the cardiovascular and central nervous system. In 1966/67 Dr. Obrez completed a cardiovascular fellowship in Radiology at Stanford in Palo Alto (under prof. Abrams). After his return to Ljubljana, he started performing coronary angiography.^14^ Obrez was highly respected by his cardiology peers and trained cardiologists Majda Mazovec, Anton Jagodic, Borut Pust, Andrej Cijan, Peter Rakovec, Darko Zorman and Darja Fettich, pediatric specialist. Dr. Fettich started children heart catheterization in Ljubljana.^14^–^17^ Ivo Obrez also trained many radiology and cardiology fellows in coronary angiography.

### Diagnostic cardiovascular angiography

**FIGURE 1.**
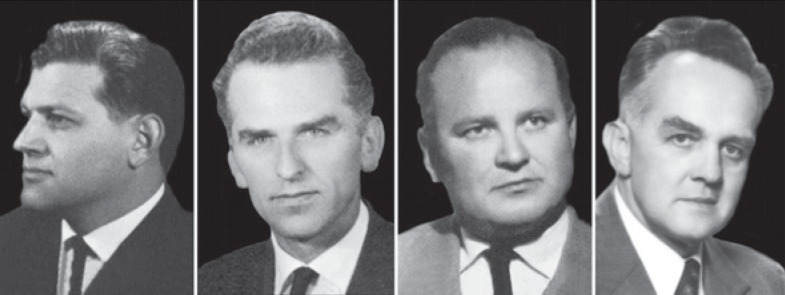
The pioneers of angiography in Slovenia. From left to right: Ivo Obrez, Jože Stropnik, Stanko Hernja and Miro Košak.

In 1960s was a time of great change in radiology. Prof. Stanko Hernja was determined that his Institute of Roentgenology would contribute to the pace of that change. His commitment included dislocated x-ray unit at the Department of Surgery for enhanced studies of the heart, central nervous system and other vessels. This cardiovascular unit, led by Jože Stropnik, has not been fully equipped with new x-ray equipment. This is why Dr. Stropnik modified homemade cassette changer (Electro-medicina, Ljubljana) for peripheral angiography using five 120 cm long cassettes. Because rotation of the cassettes occurred through the center of the focal spot, there was no blurring. Long leg angiograms showed the vascular system from abdominal aorta to the ankle.^18^ His modification has been in use for 30 years. When the Institute of Roentgenology moved to the new University Clinical Centre in 1974, this homemade modification moved with it (Figure 1).

**FIGURE 2.**
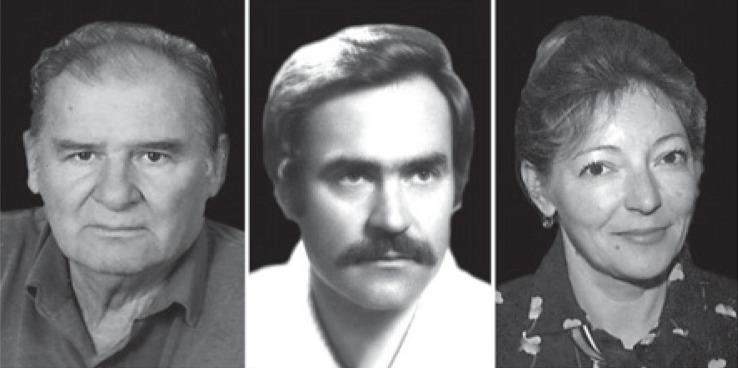
The pioneers of angiography in Slovenia. From left to right: Jože Košir, Peter Soklič and Elizabeta Baretić-Kolar.

**FIGURE 3.**
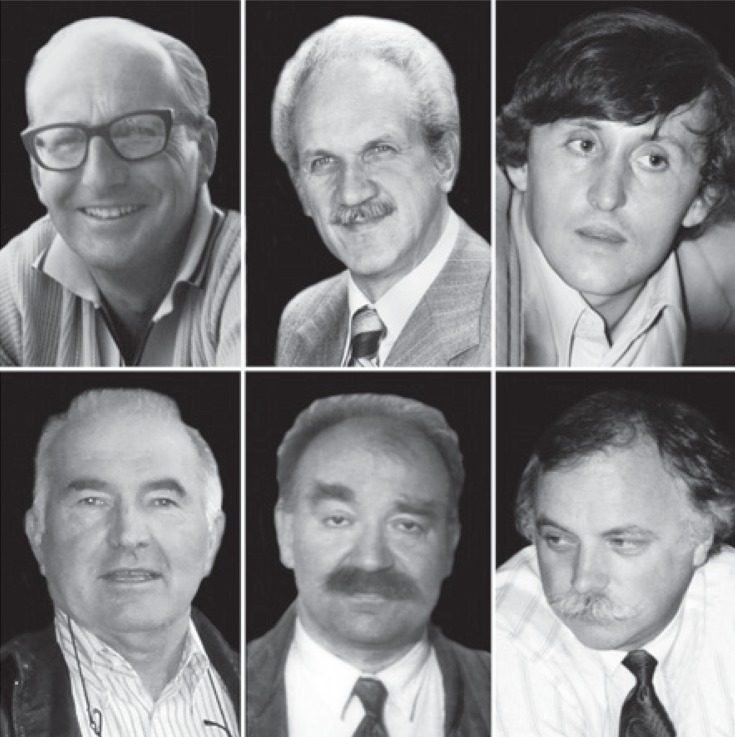
The pioneers of angiography in Slovenia. From left to right, upper row: Uroš Vizijak, Marijan Pocajt, Dušan Pavčnik. Lower row: Sead Galijaš, Dušan Tomažič and Jože Matela.

**FIGURE 4.**
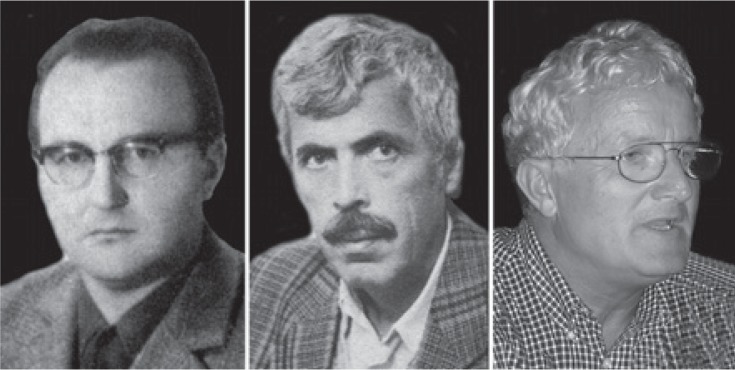
Neuroradiologists. From left to right: Martin Čerk, Tomaž Kregar and Miha Škrbec.

In 1971 Stropnik and Obrez described angiographic diagnostic examination of gastrointestinal bleeding.^19^ In early 1970s, Stropnik reported on angiographic image of the liver cirrhosis^20^ and on diagnostic importance of variations of celiac trunk.^21^ Carotid angiography was performed either by direct injection through a percutaneously inserted needle or by catheter technique. Neuroradiologist Martin Čerk reported on occlusion of internal carotid artery.^22^ Čerk, Tomaž Kregar and Miha Škrbec published their own experiences on cerebral angiography.^23^ Soklič, Stropnik, Obrez, Košir and Baretić-Kolar published the use of selective angiography in the diagnosis of intraluminal and extraluminal abdominal bleeding. They described that duodenal blood supply originates both from the celiac and superior mesenteric arteries. It was often necessary to inject each artery separately in order to demonstrate the site of bleeding.^24^ Jože Košir and Nataša Budihna compared isotope venography with conventional venogram in patients with deep vein thrombosis of the limb.^25^ Jurij Us and Jože Košir investigated the potential of the internal mammary artery angiography for the diagnosis of the breast diseases (Figure 2).^26^ Other people like Uroš Vizijak from Celje, Marijan Pocajt, Dušan Tomažic and Jože Matela from Maribor, Jože Kocijančič from Murska Sobota, and Dušan Pavčnik and Sead Galijaš from Nova Gorica have to be given credit for the development of angiography in Slovenia as well (Figures 3, 4).

## Interventional radiology

Charles Dotter, the father of interventional radiology (IR), was the first to perform angioplasty on a peripheral artery. In his most famous case, Dotter used a guide wire and Teflon coaxial catheters to dilate a superficial femoral artery stenosis in an 82 year old woman with limb ischemia and gangrene who refused amputation. He was successful, the patient was ambulatory for the remainder of her life. That event changed the practice of medicine in the world^27^,^28^; however it took quite a while for angiographers to change diagnostic thinking and to develop interventional technique and devices. In 1976, Grüntzig reported on percutaneous transluminal angioplasty (PTA) balloon catheters for iliac and peripheral arteries stenosis and in 1979 on coronary balloon catheters for coronary angioplasty.^29^,^30^

After successful experimental and clinical reports of Drs. Rösch, Baum and Nussbaum in the early 1970s, selective vasoconstrictive infusions became a useful technique for stopping both arterial and venous gastro intestinal bleeding.^31^ Rösch reported: “When we could not stop bleeding from a gastric ulcer in a coagulopathic young patient with vasoconstrictive infusions, we selectively embolized the gastro-duodenal artery with autologous blood clot”. The publication of this case together with experimental studies was the basis for the wide use of embolization for treatment of arterial gastrointestinal bleeding.^32^

The goals of selective or local thrombolysis are to relieve an acute vascular obstruction by thrombus and unmask the underlying pathology. Charles Dotter started selective thrombolysis in 1972 to treat complications of angiography and PTA.^33^

Transcatheter device technology began in the 1970s with work of Porstmann in occluding patent ductus arteriosus^34^, King and Rashkind in closing atrial septal defect^35^,^36^, and Gianturco and his associates in occluding blood vessels.^37^ In the 1980s, Palmaz and coworkers extended Dotter’s late 1960s concept^38^ by introducing balloon expandable stent, to treat stenotic vascular lesions.^39^ Caesar Gianturco conceived a spring like zig zag stent made of stainless steel and described his experimental results in 1985.^40^ The largest proliferation of device technology started in 1990s.

The transjugular intrahepatic portosystemic shunt (TIPS) is a percutaneous alternative to surgical portosystemic shunts that was conceived in the late sixties by Josef Rösch. A TIPS is a side-to-side shunt of determined diameter designed to function as a partial shunt that preserves a portion of portal flow to the liver.^41^

### IR in Slovenia

**FIGURE 5.**
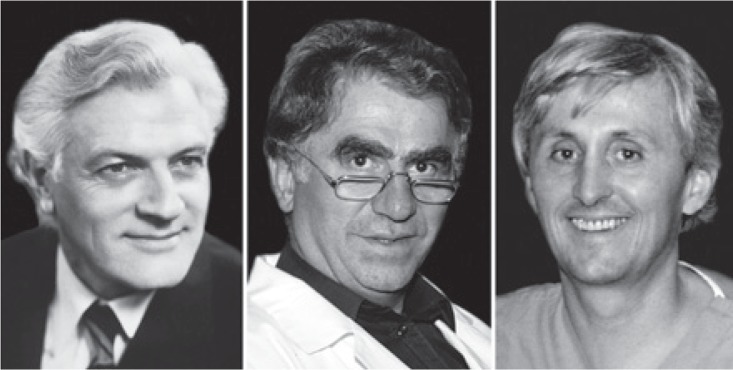
The pioneers of interventional radiology. From left to right: Ivo Obrez, Miloš Šurlan and Dušan Pavčnik.

**FIGURE 6.**
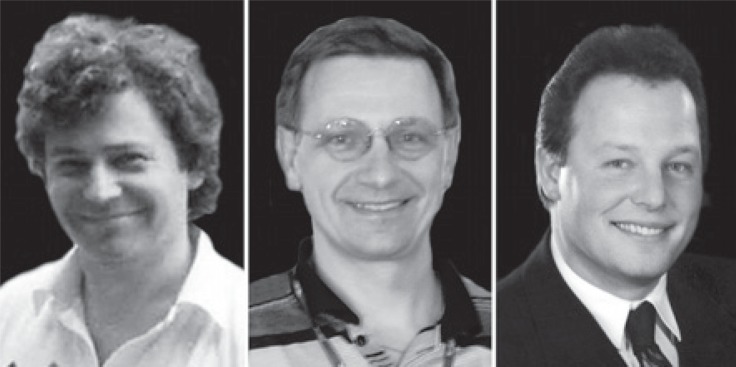
The pioneers of interventional radiology. From left to right: Janko Klančar, Pavle Berden and Jernej Knific.

**FIGURE 7.**
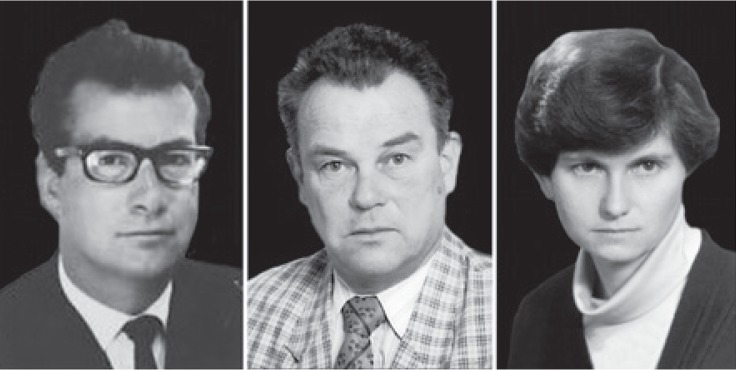
The pioneers of interventional radiology. From left to right: Marijan Jereb, Jurij Us and Erika Brenčič.

Between 1969 and 1980 Slovenian radiologists issued many reports and published papers on interventional radiology procedures. Auersperg, Us-Krasovec and Obrez introduced the use of selective intra-arterial chemotherapy and reported its complication.^42^,^43^ Obrez reported experimental study on temporary occlusion of the renal artery and its effects and significance.^44^ Obrez and Kubicka reported simultaneous infusion of vasopressin into two arteries to control massive colonic hemorrhage using a new catheter.^45^,^46^ Marijan Jereb published the usefulness of needle biopsy in chest lesions of different sizes and locations. Direct puncture technique proved to be an invaluable aid in the diagnosis of chest lesions.^47^ Jurij Us reported on aspiration biopsy of the retroperitoneal lymph nodes in 1977.^26^ In 1980, Obrez made an attempt to define the present status of IR at home and abroad. He stated that IR occupies a unique place in medicine. Obrez performed the first PTA of superficial femoral artery in 1978.^48^,^49^ In Ljubljana University Clinical Centre, Miro Košak was the head of cardiovascular surgery and we all knew it. He respected Ivo Obrez because of his knowledge, catheter skills and clinical judgment. Košak offered surgical stand by to radiologists when performing balloon angioplasty procedures including PTA of femoral, iliac or renal artery.^49^ Viktor Videčnik, Elizabeta Baretić-Kolar and Miloš Šurlan reported on thrombolytic therapy for femoro-popliteal occlusions. The lysis has been helped by streptokinase and urokinase. The underlying stenoses were treated by PTA (Figures 5, 6, 7).^50^

### Drainage of retroperitoneal and pelvic abscesses and fluid collections

You can help save a life by draining an abscess percutaneously in a septicaemic patient using image guidance and a small catheter, particularly in postoperative patients. Success of abscess drainage depends upon complete evacuation of the cavity, control of an underlying condition and prevention of reaccumulation. We published our results in 1984.^51^

### Biliary system

Obrez and coworkers reported on percutaneous biliary drainage (PBD) in 1980.^52^ Percutaneous transhepatic cholangiography was usually part of interventional procedure as percutaneous biliary drainage. There was continuing evaluation of different technique for draining the gallbladder and obstructed hepatic biliary ducts. Catheters and plastic prosthesis could serve as channels to keep open ducts that were blocked. One of common complication was replacement of dislodged catheter. However; the role of PBD was also changing with the development of interventional endoscopy.^53^

### Percutaneous urinary interventions

Relief of acute urinary obstruction was the most common intervention performed by radiologist.^54^ Percutaneous access to the kidney has also been used to remove stones, or to place antegrate ureteral stents when retrograde stenting by urologist failed.^55^ Miloš Šurlan reported treatment of renal cysts with percutaneous alcohol ablation.^56^ The development of extracorporeal lithotripsy and ureteroscopy has limited the number and variety of percutaneous procedure performed in urinary tract.

### Embolotherapy

**FIGURE 8.**
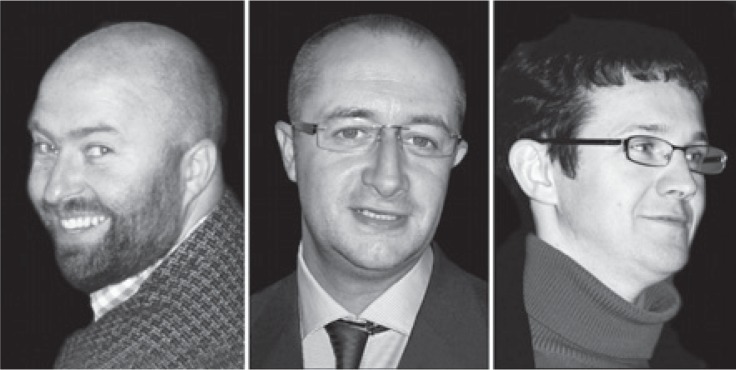
Interventional radiologists. From left to right: Tomaž Ključevšek, Dimitrij Kuhelj and Peter Popovič.

**FIGURE 9.**
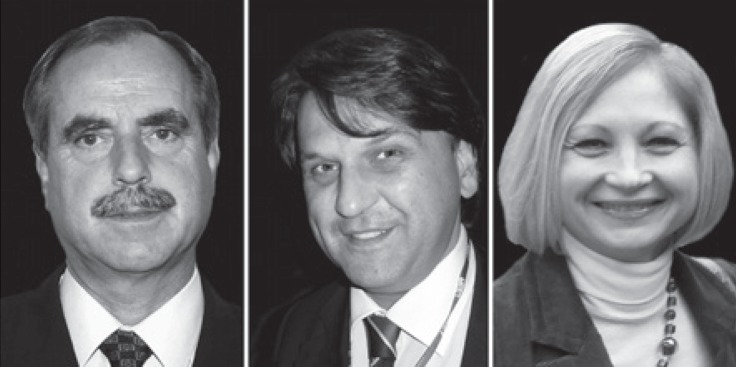
Interventional radiologists. From left to right: Tomaž Šeruga, Zoran Milošević and Vladka Salapura.

No single embolic agent is universally applicable to all clinical situations. Ivo Obrez reported the use of gelatin sponge and coils for renal tumor treatment in 1978.^57^ Prior to performing an embolization, several factors must be considered. These include the desired level of occlusion, the desired duration of occlusion, the relevant vascular anatomy and the available embolic material.^57^ Martin Čerk reported embolization of anteriovenous malformation by occluding anterior and middle cerebral artery in 1979. He used gelatin sponge.^58^,^59^ Various embolic materials have been used clinically to control hemorrhage^60^, relieve pain, inhibit tumor growth, and facilitate resection by reducing vascularity and tumor bulk. Janko Klančar described embolotherapy of kidney tumors.^61^ Other applications of embolotherapy were reported by Pavel Berden on ablation of tumors using absolute ethanol^62^, by Rok Cesar on palliative and preoperative treatment of bone tumors^63^, by Miloš Šurlan on chemoembolization of malignant liver tumors^64^ and Dušan Pavčnik on venous impotence and percutaneous embolization treatment (Figures 8, 9).^65^

### Endoluminal stenting

The era of stenting in Slovenia started with the use of self expanding Gianturco Z stents in 1989. Pavčnik and Šurlan reported that three patients with stenosis of the tracheobronchial tree and one with the obstruction of *vena cava superior* were treated with self expanding stents.^66^ While this initial results with the stents were encouraging, these stents should be viewed as a first generation device. In early 1990s we used Palmaz stent, Strecker stent and Wallstent in the arterial system including peripheral, iliac and renal arteries. We published our results recommending predilatation and those lesions have to be covered from healthy to healthy segments.^65^–^70^

**FIGURE 10.**
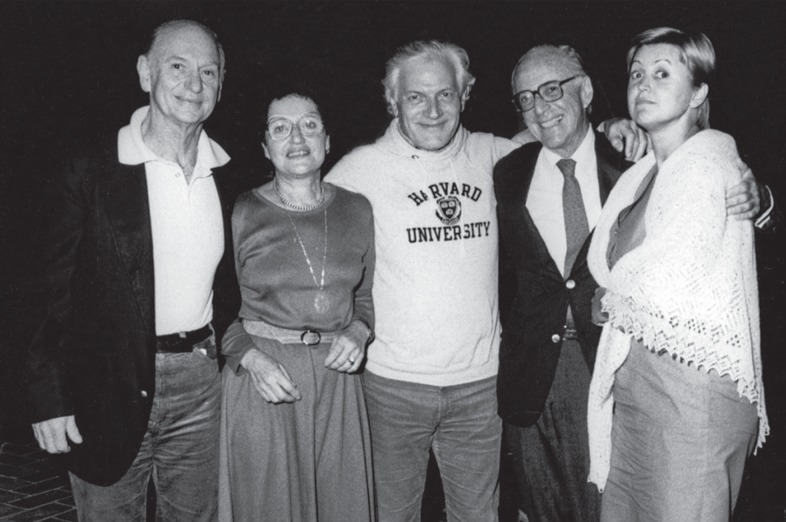
From left to right: Alexander Margulis, Mrs. Abrams, Ivo Obrez, Herbert Abrams and Hedvig Hricak on the occasion of Joint meeting and postgraduate course organized by Ivo Obrez and ECA, ESCVIR and ASCVIR in Dubrovnik in 1983.

**FIGURE 11.**
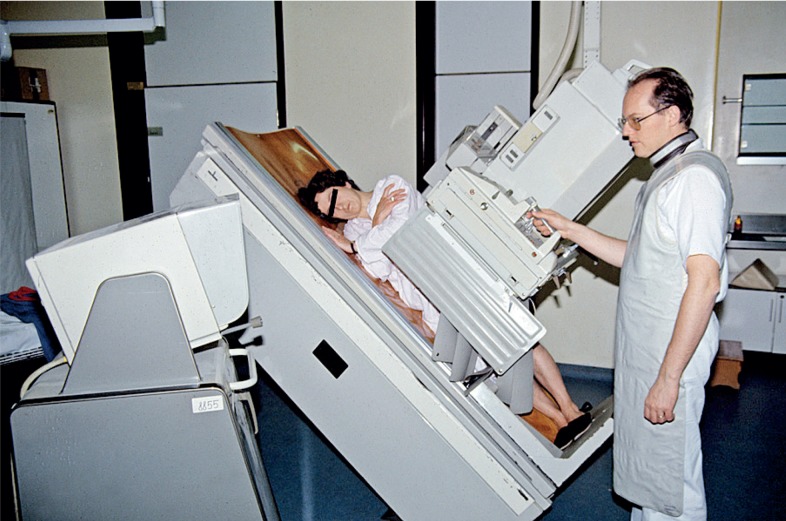
Siemens Sireskop (1974–1980s). With electronic image intensification, examination could be made in normal light and recorded on x-ray film or videotape. Maks Kadivec is performing a neuro-radiological procedure. Similar Siemens unit (next door) modified with rapid-change film holder (portable puck) allowed multiple images essential for studies of the cardiovascular system (1964 – early 1990s). There was as well modified homemade cassette changer (“boben”) for peripheral angiography.

**FIGURE 12.**
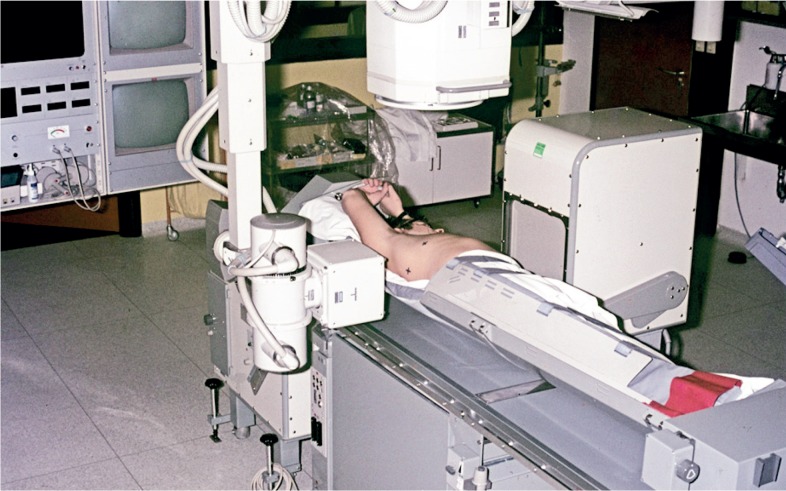
Tridoros-Siemens Elema angiocardiographic unit with Schönander rapid-change film holders (1974–1986).

**FIGURE 13.**
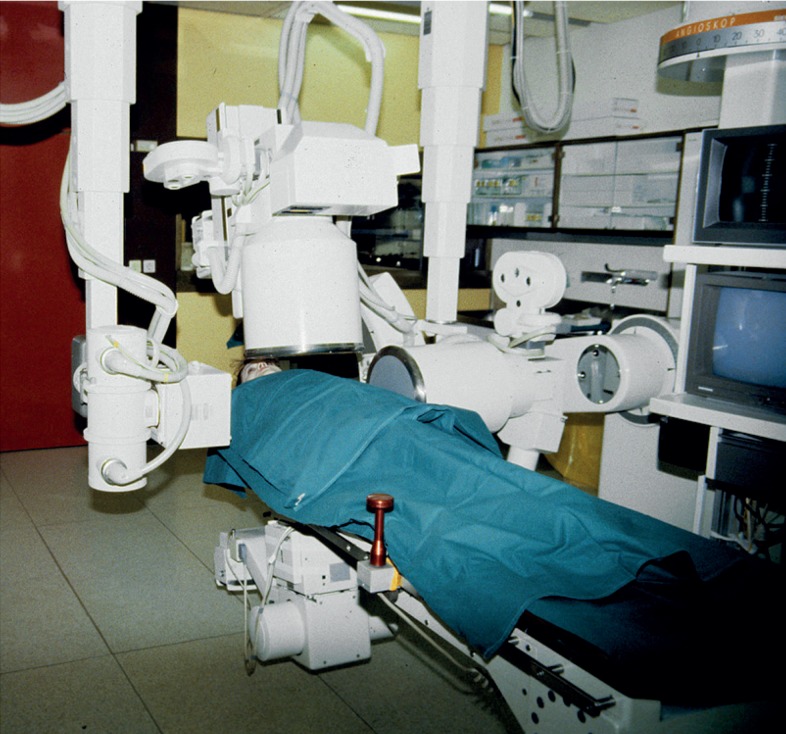
Bi-plane Angioskop for coronary cine-angiography (1986–2008).

**FIGURE 14.**
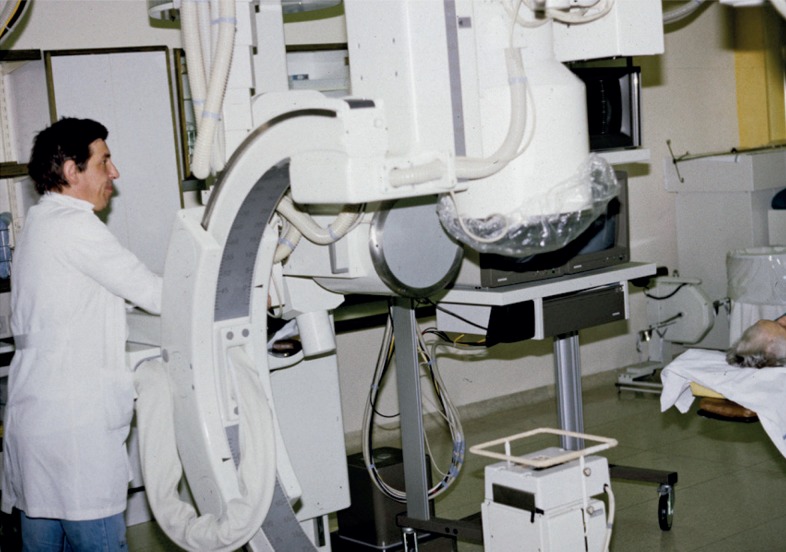
Angioskop-the state of the art bi plane Siemens C-arm digital subtraction and cine- angiography (1986–2008) (Jaka Regvart, Radiol. Ing.).

**FIGURE 15.**
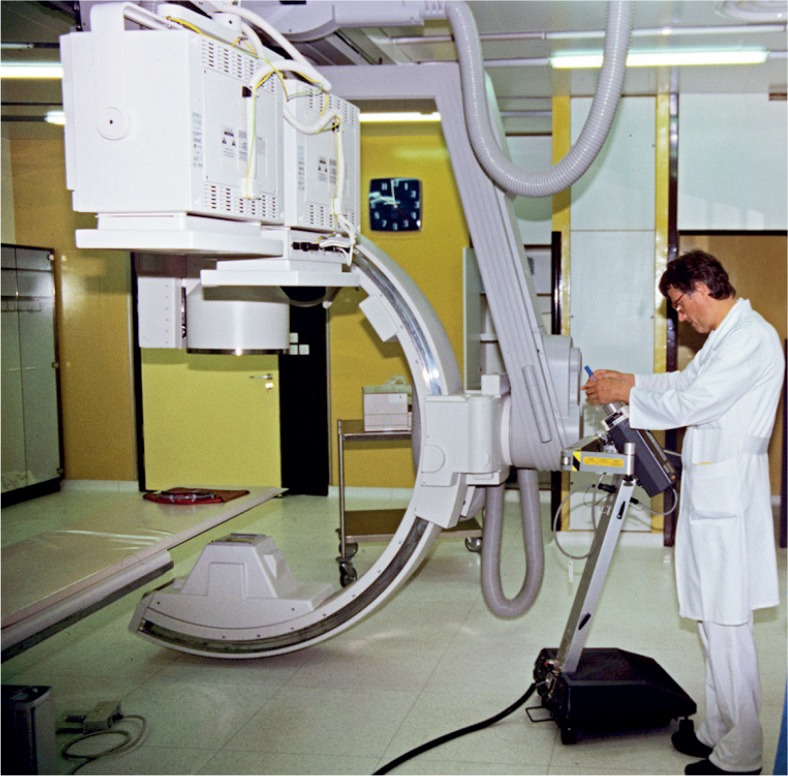
Philips-Integris C for digital subtraction angiography (1993–2009) (Marijan Klemenc, Radiol. Ing.)

In 1993, the first TIPS was created by the self expanding Wallstent in Ljubljana. Creation of this shunt resulted in a decrease of 18 mmHg in portal pressure. The main advantage of Wallstent was its extreme flexibility. Being encouraged by this result, Pavčnik and Šurlan attempted TIPS in additional patients with severe cirrhosis and variceal hemorrhage.^71^ Peter Popovič *et al.* reported TIPS versus endoscopic sclerotherapy in the elective treatment of recurrent variceal bleeding (Figures 10–15).^72^

### Heart, aorta and *inferior vena cava*

In 1979 Obrez travelled to Zurich to see Andreas Grüntzig performing coronary angioplasty. He was the first one to do percutaneous transluminal coronary angioplasty (PTCA) in Slovenia in 1980.^73^ In 1986 Institute of Roentgenology opened a new Catheterization Laboratory with the state of the art Siemens C-arm cardio angiography. For many years coronary angiography, coronary interventions and pediatric heart procedures were performed in this laboratory. We published our experience after 50 PTCA.^74^ Pavčnik and Kranjec performed PTCA of acute coronary occlusion followed by intracoronary and intravenous thrombolysis. They introduced this coronary therapy into Slovene medicine in 1989.^75^ Pavčnik, Cijan, Bricelj and Robida reported results on transcatheter balloon valvuloplasty of pulmonary, mitral and aortic valves. Authors described valvuloplasty in congenital and acquired valve disease. From 1987 to 1993 we performed thirteen pulmonary, twenty-six mitral and five aortic valve dilatations.^76^,^77^

Dušan Pavčnik, Pavle Berden and Mirta Koželj published case report of the transcatheter occlusion of patent ductus arteriosus using Rashkind double umbrella. After two years follow up our two patients were free of symptoms.^78^

In 1995 we reported two cases with inoperable descending thoracic aortic aneurysm. Both patients underwent an intraluminal bypass of the descending thoracic aneurysm with a stent graft. Stent grafts were constructed from Gianturco-Rösch stents and a soft Dacron graft at the Dotter Institute laboratory.^79^ First patient with gigantic aneurysm developed two late complications during a 7 years follow-up. He was additionally treated with two endografts.^80^ In 2010 Dimitrij Kuhelj *et al*. published risk of deterministic effects during endovascular aortic stent graft implantation.^81^

In 1994 we reported the case of young patient with two symptomatic transient ischemic attacks due to arterio-venous fistula. Patient underwent transcatheter closure of the shunt with Gianturco coils. Patient developed transient pleurisy few days after embolization. Percutaneous transcatheter occlusion of pulmonary anteriovenous fistula has become the treatment of choice replacing surgical intervention.^82^ Vladka Salapura, Tomaž Ključevšek, Dimitrij Kuhelj and Peter Popovič reported on *inferior vena cava* (IVC) filters in 2008.^83^

Tomaž Šeruga in 2004 and Zoran Milošević in 2007 reported transcatheter treatment of aneurysms in cerebral circulation using coils. Šeruga detached electrolytically detachable platinum coils.^84^,^85^ In 2010 Vladka Salapura *et al.* reported study: Infrapopliteal run-off and the outcome of femoropopliteal percutaneous transluminal angioplasty in Vasa.^86^ In 2013 Dimitrij Kuhelj *et al.* published about percutaneous mechanical thrombectomy of superior mesenteric artery embolism.^87^

## Ivo Obrez

Prof. Obrez rose through the academic ranks. He became associate professor in 1972 and a full professor in 1984. He was named director of the Institute of Roentgenology in Ljubljana in 1974. Through his book chapters, articles, lectures and trainees from Slovenia and former Yugoslavia, Dr Obrez has dominated the specialty for more than two decades. His impact on the field of angiography and IR was important not only in Slovenia and former Yugoslavia but also in Europe. He supported demand for people and equipment and, in turn, expected service and quality. He worked very closely with surgeons and his cardiology colleagues at University Clinical Center in Ljubljana. His philosophy was to try to prevent turf battles and collaborate. Dr Obrez returned to the United States as visiting professor in 1972 at Stanford, in 1978/79 and in 1981 at Harvard (Brigham and Women’s Hospital) in Boston. Ivo Obrez was the Executive Committee member when European College of Angiography (ECA) joined forces with the European Society of Cardiovascular and Interventional Radiology (ESCVIR), to form CIRSE in 1985. In 1983 Obrez was the Meeting Chairman for the joint meeting (ECA, ESCVIR and American ASCVIR) in Dubrovnik (Figure 10). Ivo Obrez died of pancreatic cancer in 1989.

## Conclusions

Author reviewed the Slovenian experience from diagnostic angiography to IR. He reviewed briefly the beginning of interventional vascular radiology and the origins of IR’s major interventional vascular and non vascular procedures. After these beginnings, many interventionalists contributed to further improvement or modifications of these procedures. In 1995, The author moved to Portland, Oregon. He is currently Director and Professor of Research at the Dotter Interventional Institute, Oregon Health and Science University. He has published over 120 scientific publications including 5 studies in Slovenian journal Radiology and Oncology.^87^–^91^

Historical data show that Slovenian radiology has relatively immediately introduced the new methods of interventional radiology in clinical practice.
